# Prototype Design and Experimental Evaluation of Autonomous Collaborative Communication System for Emerging Maritime Use Cases

**DOI:** 10.3390/s21113871

**Published:** 2021-06-03

**Authors:** Jiri Pokorny, Khanh Ma, Salwa Saafi, Jakub Frolka, Jose Villa, Mikhail Gerasimenko, Yevgeni Koucheryavy, Jiri Hosek

**Affiliations:** 1 Department of Telecommunications, Faculty of Electrical Engineering and Communication, Brno University of Technology, Technicka 12, 616 00 Brno, Czech Republic; saafi@feec.vutbr.cz (S.S.); frolka@feec.vutbr.cz (J.F.); hosek@feec.vutbr.cz (J.H.); 2 Unit of Electrical Engineering, Tampere University, Korkeakoulunkatu 7, 337 20 Tampere, Finland; manguyenquangkhanh@gmail.com (K.M.); gerasimenkoma89@gmail.com (M.G.); evgeny.kucheryavy@tuni.fi (Y.K.); 3 Mechatronics Research Group, Tampere University, Korkeakoulunkatu 6, 337 20 Tampere, Finland; jose.villa@tuni.fi

**Keywords:** autonomous vehicles, maritime use cases, USV, UAV, collaborative communication system, directional wireless links, prototype design

## Abstract

Automated systems have been seamlessly integrated into several industries as part of their industrial automation processes. Employing automated systems, such as autonomous vehicles, allows industries to increase productivity, benefit from a wide range of technologies and capabilities, and improve workplace safety. So far, most of the existing systems consider utilizing one type of autonomous vehicle. In this work, we propose a collaboration of different types of unmanned vehicles in maritime offshore scenarios. Providing high capacity, extended coverage, and better quality of services, autonomous collaborative systems can enable emerging maritime use cases, such as remote monitoring and navigation assistance. Motivated by these potential benefits, we propose the deployment of an Unmanned Surface Vehicle (USV) and an Unmanned Aerial Vehicle (UAV) in an autonomous collaborative communication system. Specifically, we design high-speed, directional communication links between a terrestrial control station and the two unmanned vehicles. Using measurement and simulation results, we evaluate the performance of the designed links in different communication scenarios and we show the benefits of employing multiple autonomous vehicles in the proposed communication system.

## 1. Introduction

Digitalization and digital transformation are shaping industries across the world. Among these, the maritime industry can benefit from the process of modernizing its existing practices to improve operational efficiency [1]. As part of the systems and solutions in which vessel operators are investing, autonomous vehicles and robotics are being utilized not only in ports and harbors, but also to aid the sea transport services in offshore areas [2]. The deployment of surface, aerial, and underwater autonomous systems open possibilities for improvements in current maritime operations and assist in several services, such as maritime search and rescue, onboard applications, navigation, and fleet management [3]. However, these benefits are conditional upon the utilization of a wireless communication system that involves these entities and supports the communications between them.

**Figure 1:**
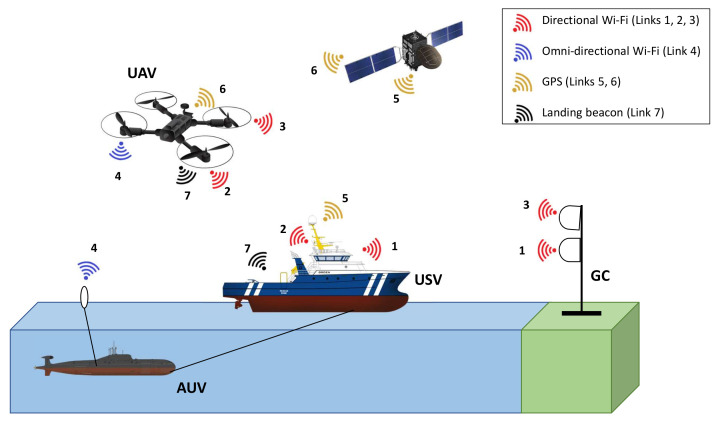
Overall layout of the proposed wireless communication system. 1. GC–USV directional Wi-Fi; 2. USV–UAV directional Wi-Fi; 3. GC–UAV directional Wi-Fi; 4. UAV–AUV nondirectional Wi-Fi; 5. USV–satellite link; 6. UAV–satellite link; 7. Beacon for UAV landing.

This trend in unmanned-vehicle-aided offshore systems is the main motivation behind launching research in this area. Specifically, the goal is to construct a prototype of a collaborative communication system composed of different autonomous vehicles operating in an offshore environment. This system is built upon surface, aerial, and underwater components represented by a Unmanned Surface Vehicle (USV), Unmanned Aerial Vehicle (UAV), and Autonomous Underwater Vehicle (AUV), respectively. As part of the final prototype, a collaborative communication system should be implemented to connect these three subsystems using different radio access technologies. The overall communication layout of the system is depicted in Figure 1. Prospective applications of the AUV include underwater mapping, environmental surveying, and identification of underwater hazards to navigation. However, mainly the USV and UAV are considered in our previous and current works on the autonomous collaborative communication system. In [4], the design of a high-speed directional communication link between the Ground Control station (GC) and USV was outlined and its performance was evaluated. To further enhance the proposed automated offshore system, alternative solutions should be taken into account to maintain the GC–USV connection in cases where the Line-Of-Sight (LOS) link cannot be established. Hence, we build upon the work in [4] by involving the aerial component represented by the UAV to complement the previously proposed GC–USV setup and, thus, to enable a fully fledged automated offshore system [5].

The rest of this article is organized as follows: Section 2 provides a description of the state-of-the-art of the industrial applications of UAVs, specifically in the maritime industry, their different communication scenarios, and the challenges of their deployment with the review of relevant related research works. In Section 3, we describe the considered wireless communication modes between the GC, USV, and UAV and discuss the communication enablers of these modes, specifically in terms of location-based beam-steering capabilities and radio propagation models. We then detail, in Section 4, the prototype architecture design of the GC, USV, and UAV with their different communication modules. Further, Section 5 describes the mechanical, power, and networking components and methods utilized in the prototype implementation of the automated offshore system. The performance of the proposed system is evaluated in Section 6, where we compare the evaluation results obtained from measurement campaigns to those obtained from simulations of selected analytical models. This comparison will allow us to identify the models that can better characterize the radio propagation in the studied scenarios and that can, thus, be used for simulation-based evaluation of future enhancements of the communication system. Additionally, the benefits of employing a UAV in the proposed communication system are evaluated on a real scenario and the technique for determining the optimal UAV location is described.

## 2. State-of-the-Art and Related Work

UAVs, commonly known as drones, are characterized by their mobility, adaptive altitude, advanced wireless communication, and sensing capabilities [6]. Such characteristics motivate several industries to employ UAVs in different operating scenarios, including but not limited to the manufacturing industry [7], agriculture [8], and public safety [9]. In fact, the global market of drones for industrial applications is projected to grow to around USD 43 billion by 2024, reaching the compound annual growth rate of 20% between 2018 and 2024 [10]. Similarly to other industries, the applications of UAVs in maritime use cases can vary from search and rescue procedures, data gathering for navigation and fleet management, and remote monitoring via video surveillance [11] to charging shipborne sensors using wireless power transfer [12].

As part of the deployment options, UAVs can be used as aerial base stations to enhance the coverage [13] and capacity of wireless networks [14]. This option can be valuable in offshore areas where LOS links with the terrestrial access network cannot be established. Additionally, UAVs can operate as flying mobile terminals and be used as relay nodes in out-of-coverage and Non-Line-Of-Sight (NLOS) scenarios [15]. In such situations, vessels can make use of the existence of proximate UAVs to access the network via relaying. In brief, UAV-assisted communications can be utilized to empower the performance of wireless networks toward reliable, massive data transmissions, and additional coverage ranges for maritime applications [16].

However, the abovementioned benefits come with various challenges in the deployment of UAVs. These challenges are in the scope of several research works, such as air-to-ground channel modeling [17], trajectory planning for UAVs performing exploration tasks in disaster scenarios [18], dynamic placement of relay UAVs [19], and the challenge of energy-efficient operation in UAV networks due to the limited onboard energy [20,21]. To tackle some of these challenges, certain research works have proposed the use of Software-Defined Networking (SDN) for several control functions in Flying Ad Hoc Networks (FANETs), including but not limited to topology management and collision avoidance [22,23].

Although involved in various applications, UAVs are utilized as the only type of unmanned vehicle in most of the systems studied in the reviewed research works. In this article, we propose the use of two types of unmanned vehicles, namely, USV and UAV, in a collaborative communication system for maritime use cases. We detail the UAV deployment scenarios in the proposed autonomous communication system in the following sections.

## 3. Proposed Communication Modes and Solution Components

**Figure 2:**
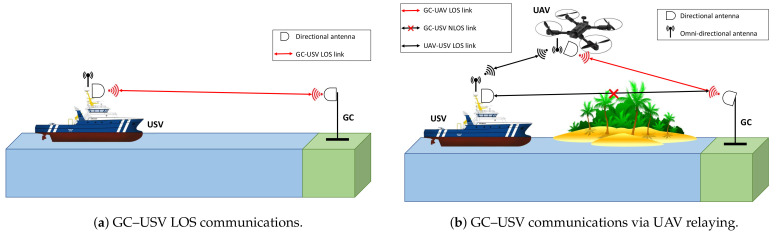
Illustration of the proposed communication modes in the autonomous collaborative system.

Based on the LOS/NLOS scenarios, two communication modes between the GC and the USV in the presented automated offshore system are proposed, namely, a LOS communication mode and a relay communication mode. The first mode is based on LOS communications between the GC and the USV and is depicted in Figure 2a. The second communication mode utilizes relaying via UAV in an NLOS scenario, as demonstrated in Figure 2b.

To establish high-speed links, the GC, USV, and UAV are equipped with directional antennas and dynamic beam-steering capabilities. In Section 3.1, the location-based beam-steering algorithm utilized in our communication system is introduced. In addition, propagation models are utilized to predict the channel path losses and, thus, to characterize the behavior of radio propagation in the communication system with the consideration of certain environment effects. Further details on the selected propagation models are provided in Section 3.2.

### 3.1. Location-Based Beam-Steering Algorithm

As depicted in Figure 2, the utilization of directional antennas depends on the communication mode. In the LOS communication mode, both the USV and GC are equipped with directional antennas. In order to align the beams of these antennas in the system, an algorithm for beam-steering based on devices’ locations was developed in [4]. This algorithm included two main steps: (1) calculation of the actual beam-steering angle; (2) transmission of the control commands with the new angle value.

In the relay communication mode, both the USV and the UAV are equipped with one directional antenna and one omnidirectional antenna while the GC carries one directional antenna. Using the same algorithm proposed in [4], the drone’s yaw can be set in a way that the installed antenna is horizontally aligned with the GC. For tilting of the antennas on the UAV and the GC, Equation (1), obtained from [24], can be used to calculate the elevation angle between two points with elevations elev1 and elev2 above the ground:

(1) $$Elevation\;angle=\left(\frac{180}{\pi }\right)\left(\frac{elev2-elev1}{d}-\frac{d}{2R}\right),$$

where *R* is the Earth’s radius and *d* is the distance between two points (lat1, lon1) and (lat2, lon2), which can be calculated using the following Haversine formula [24]:

(2) $$d=2r\sin^{-1}\left(\sqrt{\sin^{2}\left(\frac{lat2-lat1}{2}\right)+\cos\left(lat1\right)\cos\left(lat2\right)\sin^{2}\left(\frac{lon2-lon1}{2}\right)}\right).$$

Finally, to find the tilting for the directional antennas on the UAV and the GC, the horizontal angle calculation part in the algorithm proposed in [4] should be replaced by the elevation angle using Equation (1).

### 3.2. Radio Propagation Models

The choice of radio propagation models in the presented communication system depends on the radio links in both LOS and relay communication modes, namely, GC–USV and GC–UAV–USV links. Specifically, the Free Space Path Loss (FSPL) model is used for the simulation of the communications between the UAV and the other devices. The GC–USV link can be characterized with LOS communications in the near sea-surface environment. The selected propagation models are adjusted for the 5 GHz frequency bands since the IEEE 802.11ac standard is utilized as the connectivity solution in this communication system. On top of the offered capacity and coverage, the choice of Wi-Fi technology can be justified by its low cost and high availability that make it suitable for our experimental prototyping.

#### 3.2.1. FSPL Model

The FSPL model describes an ideal radio condition between a transmitting and a receiving antenna, where only LOS link exists without other sources of scattering, diffraction, or reflection [25]. It is one of the commonly used models for characterization of the ground-to-air radio propagation [26]. Therefore, this model is used to determine path loss in the communication from surface devices to the UAV based on Equation (3) [25].

(3) $$L_{FSPL}=32.44+20log_{10}\left(f_{c}\right)+20log_{10}\left(d\right),$$

where

$L_{FSPL}$—free space path loss (dB);
$f_{c}$—carrier frequency (MHz);
*d*—distance between transmitter and receiver (km).

#### 3.2.2. Near Sea-Surface Propagation Models

To characterize the radio propagation over the GC–USV link in the near sea-surface environment, the channel model from the study of Lee et al. [27] is utilized. Authors in [27] proposed a combination of 2-ray and 3-ray propagation models and showed that the utilization of each model depends on a break point $d_{break}$. This metric indicates the transition point between the two models. In detail, the path loss prediction ability of the 2-ray model in a near sea-surface LOS environment deteriorates as the propagation distance increases beyond $d_{break}$. In this latter case, the 3-ray model becomes a better option for the path loss prediction. The break point $d_{break}$ can be estimated using Equation (4), where λ is the wavelength in meters, and $h_{t}$ and $h_{r}$ are the transmitting and receiving antenna heights in meters, respectively [27].

(4) $$d_{break}=\frac{4h_{t}h_{r}}{\lambda }.$$

**Figure 3:**
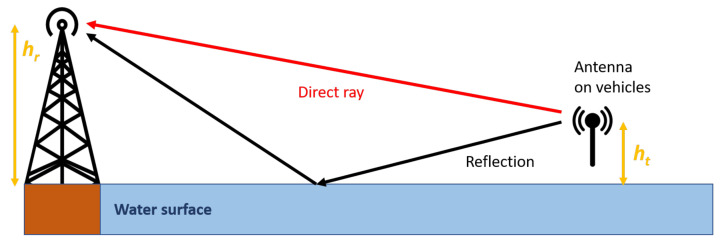
Representation of the 2-ray radio propagation.

The representation of the utilized 2-ray path loss model is depicted in Figure 3 and the predicted path loss $L_{2-ray}$ (dB) can be calculated using Equation (5).

(5) $$L_{2-ray}=-10log_{10}\left\{{\left(\frac{\lambda }{4\pi d}\right)}^{2}{\left[2\sin\left(\frac{2\pi h_{t}h_{r}}{\lambda d}\right)\right]}^{2}\right\}.$$

**Figure 4:**
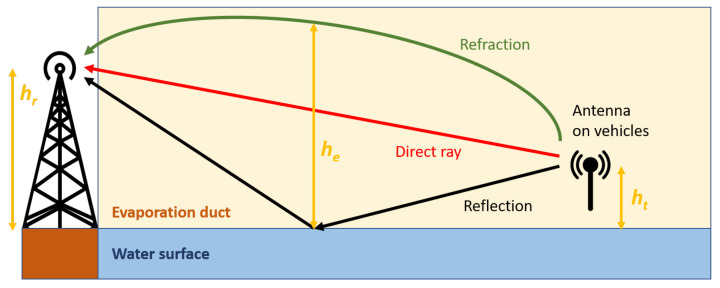
Representation of the 3-ray radio propagation.

On top of the direct and reflected rays, the 3-ray model takes into consideration the refracted ray caused by the ducting effects, as shown in Figure 4. This model assumes that the evaporation duct layer is horizontally homogeneous and $h_{e}$ is its effective height. Based on Equations (6) and (7), the 3-ray path loss $L_{3-ray}$ (dB) can be predicted.

(6) $$L_{3-ray}=-10log_{10}\left\{{\left(\frac{\lambda }{4\pi d}\right)}^{2}{\left[2\left(1+\Delta \right)\right]}^{2}\right\},$$

with

(7) $$\Delta =\sin\left(\frac{2\pi h_{t}h_{r}}{\lambda d}\right)\sin\left(\frac{2\pi \left(h_{e}-h_{t}\right)\left(h_{e}-h_{r}\right)}{\lambda d}\right).$$

## 4. System Prototype Architecture

**Figure 5:**
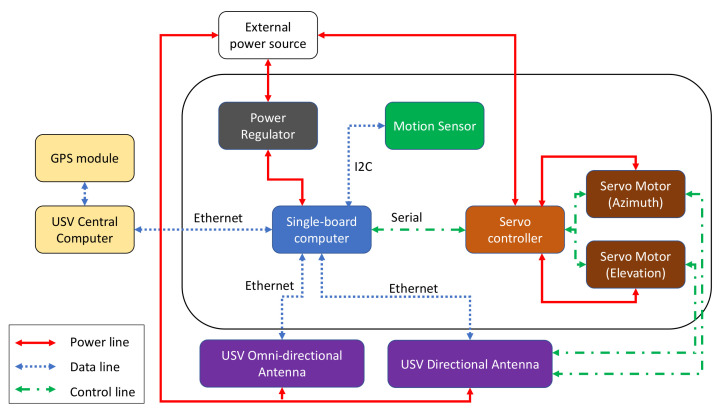
Architecture design for the USV.

**Figure 6:**
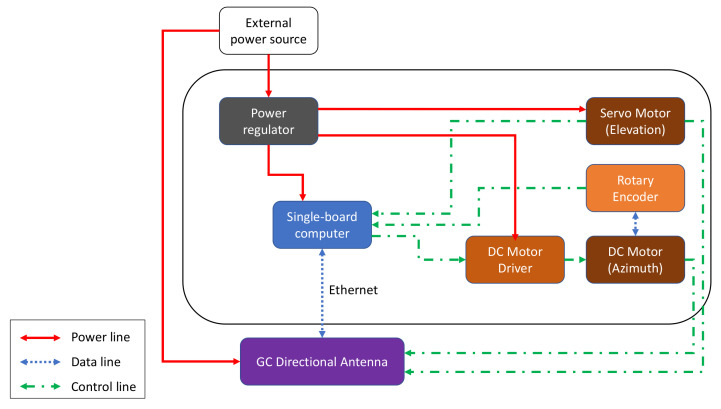
Architecture design for the GC.

**Figure 7:**
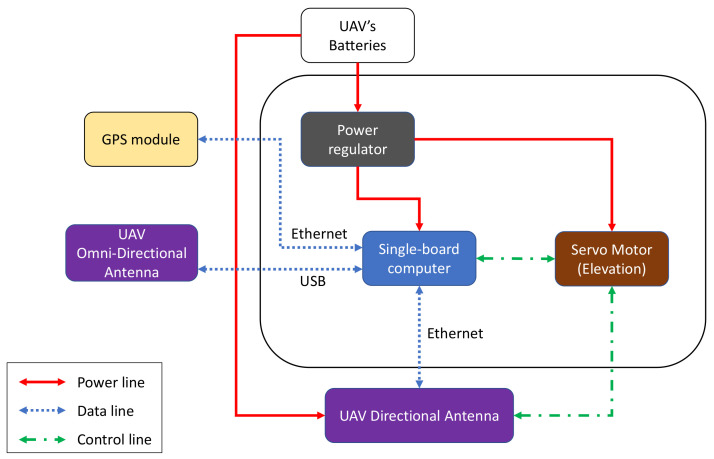
Architecture design for the UAV.

To enable LOS and relay communication modes, the communication modules of all three platforms (USV, GC, and UAV) must be equipped with long-range high-throughput transceivers. The long-range aspect can be enabled with dynamic steering of directional antennas. Each module combines various mechanical, networking, and electrical components to enable the antenna steering capability. The USV, GC, and UAV have communication modules with different architecture designs that are better depicted in Figure 5, Figure 6 and Figure 7, respectively.

The USV consists of a single-board computer, a motion sensor, two servo motors, and a servo controller installed on the compound plate together with the directional antenna. These two servo motors are responsible for the vertical and horizontal steering of the antenna. For horizontal beam-steering of the USV antenna, the positioning data (i.e., position and orientation) is collected from a Global Positioning System (GPS) compass module by the USV main computer using Robot Operating System (ROS). Subsequently, the data from the GPS compass is sent to the single-board computer via Ethernet. By assuming that the location of the GC is fixed, this computer calculates the steering angle by using the positions of the vessel and the GC, then sends a control signal in the ROS format to the servo controller. The controller then activates a servo motor to rotate the USV antenna towards the GC. When the transceivers on both sides are connected through a Wi-Fi link, the USV’s GPS coordinates are sent to the ground system and collected by the controller board in GC. The GC steering system also includes a DC motor, a motor driver, and a servo motor. The DC motor steers the antenna horizontally to the direction of the autonomous vessel.

The antenna systems on both the USV and GC are capable of vertical rotation. Since the vessel is floating on water, the tilting angle of the antenna may vary. To solve this, a 6-axis sensor is utilized to estimate the compensated vertical angle. The embedded computer controls the other servo to rotate the antenna in order to keep it stable against the waves. The servo motor in the GC system steers the antenna toward the flying UAV in the case when the system works in relay communication mode.

In NLOS scenarios, a UAV can be deployed to establish a relay link. The UAV steering system includes an embedded computer and a servo motor. The computer receives GPS coordinates of the UAV, including latitude, longitude, and altitude, to calculate the tilting angle. Then, it controls the servo to steer the antenna vertically. For horizontal rotation, the yaw-control capabilities of the drone are used to keep the antenna constantly directed toward the GC. All the subsystems have a separate power regulator to provide a suitable power supply to each component.

## 5. System Prototype Implementation

**Figure 8:**
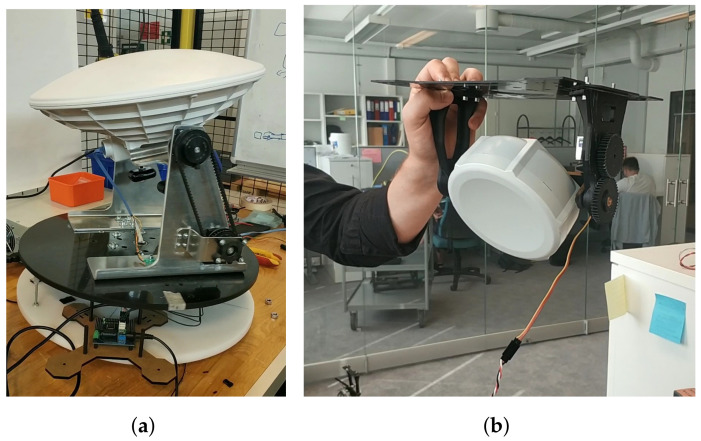
Final mechanical design of both antennas on the USV and UAV. (**a**) Mechanical platform for the directional antenna on the USV; (**b**) Mounting platform for the directional antenna of the UAV.

The mechanical structure for the communication part of the USV and GC was designed in our previous work [4]. The design of the antenna rotation mechanism for the USV is described in detail in [28]. In this work, the USV communication system was modified by using more rigid materials and more precise steering parts to improve the resistance against the wind and to increase the precision of rotation, as shown in Figure 8a. Two aluminum brackets were used to hold the antenna. This prevents the antenna from swinging when the wind is strong or when the autonomous vessel is moving at high speed. Two gears were placed on the bracket for the vertical rotation. One was connected to the servo motor through a shaft and the other was installed on the shaft of the antenna. A belt linked those two gears to transmit the rotary motion from the servo motor to the antenna. In this frame version, two ROBOTIS Dynamixel MX-28 servo motors were used for horizontal and vertical rotation of the vessel antenna. The MX-28 servo features the tracking capabilities of its speed, temperature, shaft position, voltage, and load. The shaft position can be maintained and modified accordingly for each individual servo with the control algorithm on the AX-12 actuator. It allows controlling the motor’s response in terms of speed and strength. The servo’s built-in microcontroller manages the control of sensors and antennas. The servo produces a high stall torque of 1.5 Nm and high no-load speed of 60 RPM, which are suitable for beam-steering rotation.

The directional antenna on the UAV was attached to a carbon-fiber platform. The design of this frame followed the structure of the UAV so that it could be mounted onto it, as demonstrated in Figure 8b. This mounting platform consists of several plates and has enough space to place other electrical devices on it. The brackets holding the antenna and the gears were 3D printed. The servo motor shaft was connected to one gear and the antenna was installed to the adjacent gear, which enabled the vertical rotation.

### 5.1. Single-Board Computers and Microcontrollers

The USV and GC are both controlled by the Beaglebone Green single-board computers. They are responsible for exchanging GPS messages, running beam-steering algorithms and executing measurement scripts. Table 1 shows the hardware specification of the Beaglebone Green board.

The UAV system is controlled by the Udoo X86 ULTRA version single-board computer. One task of the controller on the UAV is forwarding the network packets in the relay communication mode. The Mikrotik SXT AC antenna and the Wi-Fi USB dongle were, respectively, connected to the Ethernet and USB ports of this board. Udoo X86 was selected due to its Gigabit Ethernet network interface and USB 3.0 to provide a high data-transfer rate. In addition, the chosen embedded board is compatible with Arduino 101 platform so that it can control the servo motor using Arduino software. Besides, this board has powerful hardware that can be utilized in other autonomous tasks such as video processing. The technical specifications of the Udoo X86 board are presented in Table 2.

Another controller used in the system was the Arbotix-M Robocontroller. This robot controller is an advanced solution for Dynamixel servos and other high-accuracy robotic actuators. It incorporates a robust AVR microcontroller, a wireless interface, dual motor drivers, and 3-pin headers for hobby servos with digital and analog I/O. The Arbotix-M Robocontroller is for controlling the two, newly installed Dynamixel MX-28 servo motors.

### 5.2. Power Management

**Figure 9:**
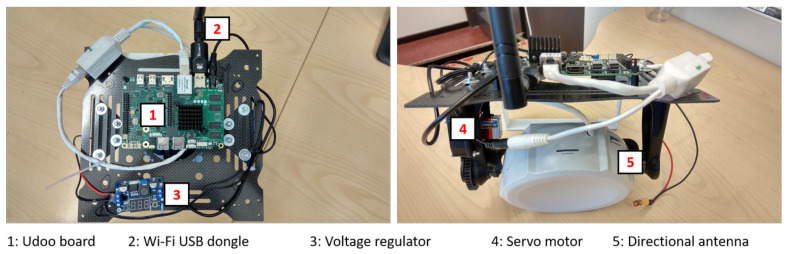
Location of components in the UAV communication system.

The power supply input varies for all the devices of the system. Thus, it is necessary to install different power regulators for each specific device. For the USV system, power was supplied by the ship accumulator and connected to the voltage regulators in the communication module of the moving vessel. The components on the UAV were provided with the power from the UAV’s battery. In order to show the power demands of utilized equipment, an overview on the input power requirements of each component is presented in Table 3. The GC and USV power supply systems have not changed from the previous design except for the installation of AX-12 servos and the Arbotix-M Robocontroller, as shown in Figure 8a. The final electrical design of the UAV is presented in Figure 9.

### 5.3. Networking Implementation

The wireless interfaces on the USV and GC were based on MikroTik DynaDish 5 devices, which support the IEEE 802.11a/n/ac (5 GHz) standard. This product is typically designed to establish a reliable point-to-point connection. Regarding the UAV system, the preferences were on lightweight devices, such as the compact MikroTik SXT 5 ac—which utilizes the same communication standard as DynaDish 5. However, the SXT 5 model comes in a small size of 140 × 140 × 56 mm and a weight of 265 g. All the chosen MikroTik devices offer Gigabit Ethernet and are compatible with Power over Ethernet (PoE).

In addition, MikroTik GrooveA 52 ac omnidirectional antennas were installed in the system. The interfaces use the same standard for wireless communication as the aforementioned equipment, and come with a Dual Band 2.4/5 GHz omnidirectional antenna with a gain of 6 dBi for 2.4 GHz and 8 dBi for 5 GHz. The interface installed on the UAV side was based on TECHKEY USB 3.0 Wi-Fi Dongle. It is a compact device with a 5 dBi antenna, which can offer up to 867 Mbps in Wi-Fi IEEE 802.11ac. It uses USB 3.0 as an interface to the controller board on the UAV.

Two routers were utilized on each side to interconnect all the devices on the USV and GC sides. The core requirements for the routers are to provide Gigabit Ethernet and support PoE for connected devices. The MikroTik hEX PoE router was installed and tested during previous stages of this work and it proved to serve well for these purposes.

### 5.4. Method for Tilt Compensation of the USV Antenna

To detect the tilting of the directional antenna on the vessel caused by waves, the Adafruit LSM9DS0 sensor was installed on the USV. This component incorporates a 3-axis accelerometer, gyroscope, and magnetometer. The Beaglebone board calculates the tilting angle, based on data collected by the LSM9DS0 sensor, and then rotates the antenna vertically to compensate the tilting. The appropriate tilt compensation software was written in Python. The Numpy and LSM9DS0 libraries were used for mathematical functions and sensor data recording, respectively. The input parameter was the current horizontal angle of the antenna. The pitch and roll values of the vessel were read from the LSM9DS0 sensor and used to define the plane in which the antenna was positioned. After that, a vector from the rotation angle values of the antenna was formed. Finally, it was possible to calculate the pointing direction of the antenna in the xyz-plane. The tilting angle was the angle between that vector and the defined plane in the xyz coordinates. After the horizontal angle is calculated, the antenna is steered vertically against the calculated angle above to compensate the vessel plane tilting.

### 5.5. GPS and Control Message Exchange Methods

**Figure 10:**
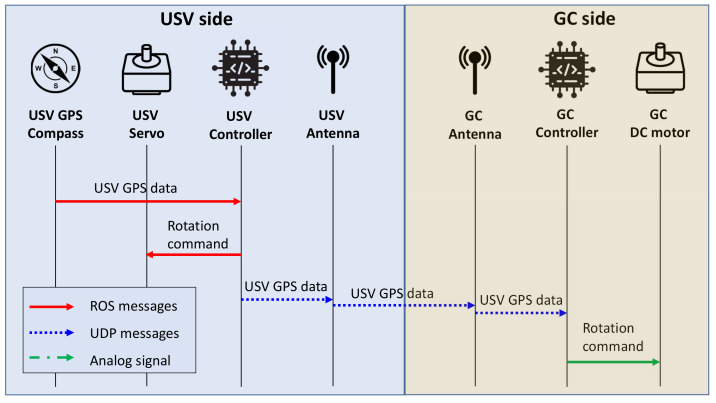
Signaling message exchange steps between the USV and GC.

The single-board computer on each vehicle is responsible for running the beam-steering algorithms. It requires the location data to calculate the rotation angle and then sends commands to specific motors. Hence, a method for transferring GPS coordinates and controlling messages among the devices in the system was designed. The diagram in Figure 10 depicts the data and control message exchange steps between USV and GC.

Since the GC was placed at a fixed location, its GPS coordinates were assumed to be known on the USV side. Before the vessel departed, the single-board computer on the USV obtained its current location from the GPS compass through ROS. The controller calculated the rotation angle from the coordinates of the vessel and the GC; then, it sent the command to the servo motor so that the USV directional antenna was pointing towards the GC. As the two antennas became aligned, the USV controller transferred the location data of the vessel to the GC side. These two embedded computers used two antennas to exchange these messages through User Datagram Protocol (UDP). When the GC controller acquired the GPS data of the vessel, it calculated and sent a rotation command as a pulse-width modulated signal to the DC motor. These steps were repeated as the USV was traveling along its route.

## 6. Experimental Results and Performance Evaluation

This section presents the results of measurement campaigns on the two communication modes, previously described in Section 3: LOS communication mode (GC–USV link) and relay communication mode (GC–UAV–USV links). Obtained measurement results from the LOS communication mode are compared to the simulation results using the near sea-surface path loss model discussed in Section 3 and proposed in [27]. Measured results from the relay communication mode serve as a motivation for UAV positioning analysis, which is described in the further part. The goal of the analysis is the investigation of the UAV’s optimal position between GC and USV. The data from both the measurements and the executed simulations were processed by Matlab.

### 6.1. LOS Communication Mode

The main idea of this first scenario is to observe how well the communication system operates in near sea-surface environment using the beam-steering algorithm for mechanical components on the USV side. The Received Signal Strength (RSS) and throughput at the USV directional antenna were recorded to evaluate the performance of the GC–USV link. The goal is to present the autonomous features of the USV and its high-speed communications with the terrestrial station.

#### 6.1.1. Measurement Scenario

The measurements were performed at the Pyhäjärvi lake, to which the Viikinsaari island is located nearby. The communication module of the USV was installed on the moving vessel and the GC system was placed at the harbor. Two laptops were connected to the directional and omnidirectional communication interfaces for running scripts to collect RSS and throughput values. A predefined route for the vessel was set between the harbor and around the island. Such a setup provided results of the system in both LOS and NLOS cases.

**Figure 11:**
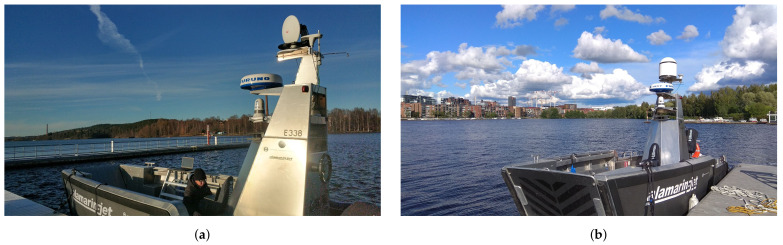
Photo of the USV with installed communication equipment. (**a**) Final implementation of the USV without the dome on the directional antenna; (**b**) Final implementation of the USV with the dome on the directional antenna.

The autonomous vessel with installed equipment is shown in Figure 11. This vessel was provided to the research group by Alamarin-Jet Oy. The directional antenna was installed on the top mast of the vessel. Additionally, a dome was built to cover the USV’s directional interface. This dome was used to protect the device from weather conditions such as rain, snow, or humidity. Besides communication devices, the USV also carried other components on the mast including a marine radar, surveillance devices, and thermal cameras, which were used to enable the autonomous capabilities of the USV.

**Figure 12:**
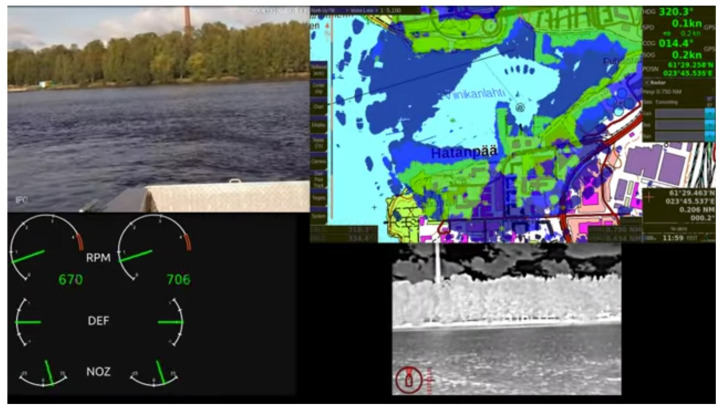
Stream from the moving vessel with telemetry data.

During the measurements, streams of data were captured by the GC containing the vessel telemetry and live camera feeds. The received data by the GC are shown in Figure 12. The top left area shows the view of the surveillance camera while the bottom right video is from the thermal camera. The record from marine radar is in the middle top of the screen. The rest shows the operation parameters of the USV.

**Figure 13:**
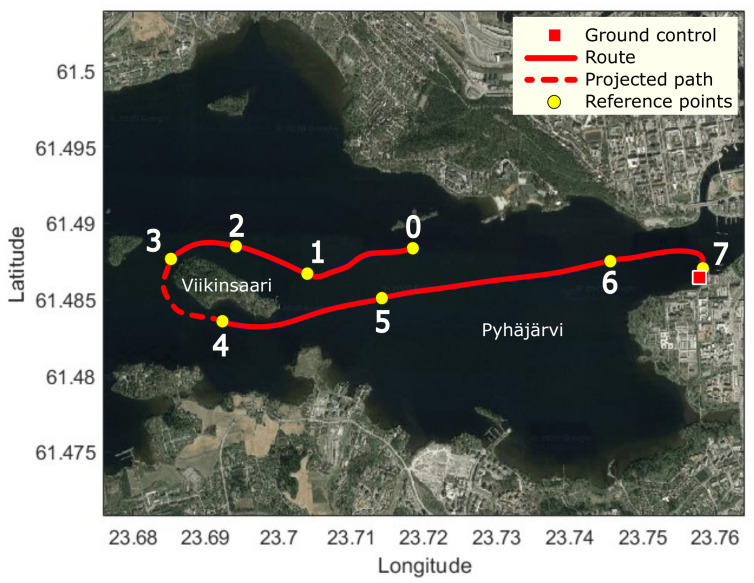
Route taken by the vessel in the LOS communication mode.

The route followed by the vessel during the measurements is shown in Figure 13. The red circle indicates the location of the GC. The GC was equipped with omnidirectional antenna installed on a tripod for the simplicity purpose. It was placed on the deck of the nearby harbor. The blue line was the actual route of the USV, which had been plotted by collecting GPS data during the route. The yellow reference points represent the exact positions of the USV, at which the RSS and throughput values were recorded and will be shown in the following plots. The collection of data started at position 0. The USV moved to the island then circled around it before going back to the GC. The paths from position 0 to position 3, and between positions 4 and 7 were characterized by LOS links between the USV and GC antennas. However, since there was an NLOS case between positions 3 and 4, the connection between the USV and GC was lost because of the island serving as an obstacle.

#### 6.1.2. Measurement Results

The aim of the measurements was to record the RSS levels and throughput values. In the baseline scenario, the route was set between the GC and the island. In the following text, the route and the results are discussed in detail.

**Figure 14:**
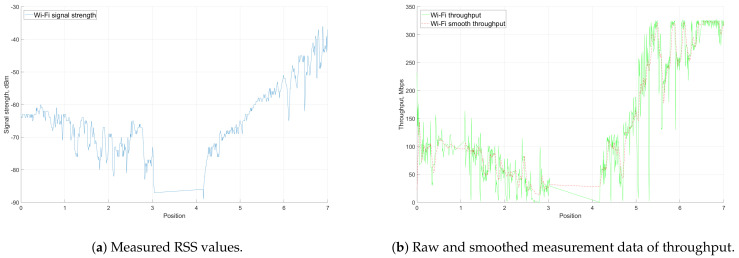
Measurement results from the LOS communication mode.

Figure 14 shows the RSS and throughput results from the measurements. The positions on the x-axis indicate the corresponding yellow points illustrated in Figure 13. Figure 14a focuses on the RSS levels at the directional antenna on the USV. As the USV moves farther away from the GC, the signal level generally decreased (positions 0–3). The RSS values fell from −60 dBm to −80 dBm during this period of time. When the vessel was moving around the island, the LOS link was blocked. There was no connection between the GC and USV, and the RSS immediately dropped below −90 dBm. The connection was re-established after the USV traveled to position 4. The RSS level started to gradually increase as the USV was approaching the harbor. The values between positions 4 and 5 were similar to those of the first part of the measurement as these positions were at approximately equal distances to the GC. In addition, as it can be seen from the graph, there were considerable fluctuations in the plot. This was mainly due to the beam-steering algorithm, mechanical stability of the antenna affected by the weather conditions, and increased speed of the USV.

Figure 14b shows the throughput results measured by the iPerf software. As the recorded throughput values fluctuated drastically, the smooth throughput showed the changing trends of the measurement results. The changing pattern of the plot was similar to the RSS level graph—decreased from position 0 to 3; dropped to 0 Mbps between positions 3 and 4; and finally, increased between positions 4 and 7. Generally, the higher signal level connection provided better throughput. When RSS was higher than −60 dBm, the throughput was higher than 150 Mbps. The signal level higher than −50 dBm may make the throughput go up to over 300 Mbps. When the USV lost the LOS link (between positions 3 and 4), there were no packets transferred between the GC and the USV due to the session interruption caused by the NLOS.

#### 6.1.3. Simulations and Evaluation

Section 6.1.2 presented the measurement results in the LOS communication mode between the GC and the sea-surface vehicle. In this section, these results are compared to simulation results of the analytical near sea-surface propagation model described in Section 3.2.2.

**Figure 15:**
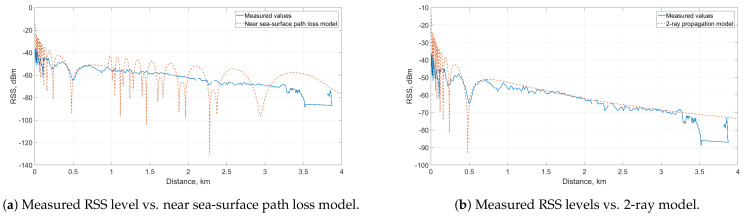
Measured RSS values in LOS communication mode compared to near sea-surface and 2-ray models.

In order to get the performance metric of interest (i.e., RSS), the $P_{tx}+G_{tx}-PL+G_{rx}$ formula was used. First, the path loss was calculated using Equations (4)–(7). As the practical installation of the devices, the height of the transmitter (omnidirectional antenna on the GC) $h_{t}$ was 2 m, while the height of USV antenna $h_{r}$ was 3 m. For the duct layer, the study of Lee et al. [27] was followed and the obtained values were used. During the LOS communication mode measurements, the transmission power $P_{tx}$ was set to 11 dBm, combined with a receiver antenna gain $G_{tx}$ of 23 dBm, which is the maximum Effective Radiated Power (ERP) in Finland. The receiver, which is the directional antenna on the USV, has a gain of 23 dBi assuming that the main beam of the antenna points to the transmitter. The resulting RSS of the near sea-surface channel model along with the measured data is illustrated in Figure 15a.

The blue data line depicts the results from the LOS communication mode measurements (specifically, when the USV is moving from the island to the harbor), which has been plotted against the distance between GC and USV. The drop at 3.5 km indicates the loss of connection between the two antennas when the USV moved behind the island. As described in Section 3.2.2, the RSS level calculated from the theoretical model in [27] has two parts separated by the break point. The distance $d_{break}$ separates the 2-ray (distances from 0 to $d_{break}$) and 3-ray (distances from $d_{break}$ further) path loss models. In the LOS communication scenario, the $d_{break}$ is at approximately 1 km. The simulated RSS values plotted according to the near sea-surface model follow the measured values approximately until the $d_{break}$ distance. After this break point, the simulation results have a different pattern. This suggests that the 2-ray model can better characterize the behavior of radio propagation in this scenario.

Another plot was generated with the 2-ray model in Figure 15b to confirm the previous conclusion. The 2-ray model very closely follows the trend of the measured values. At some points, values obtained during the measurements were lower than in the simulations, which could have been caused by weather conditions.

### 6.2. Relay Communication Mode

After measurement of the LOS communication mode between the GC and the USV, this section focuses on the relay communication mode, where UAV serves as the relaying node. The core idea of this measurement is to prove the feasibility of a relay between the GC and USV in scenarios where line of sight is not available, i.e., the route between points 3 and 4 in the previous scenario.

#### 6.2.1. Measurement Scenario

**Figure 16:**
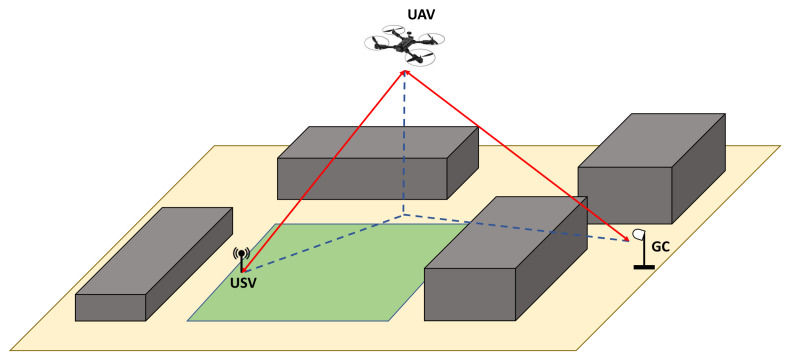
UAV relay scenario in Tampere University, Hervanta campus.

It was not possible to conduct the measurements in the same lake scenario as that utilized in the LOS communication mode due to the scenario complexity and UAV flight range. For that reason, a proof-of-concept measurement was conducted in a university campus; the scenario is illustrated in Figure 16. In the area, typical obstacles such as buildings and vehicles were present. These obstacles can simulate structures on islands or other vessels when a water surface scenario is considered. During the measurements, the GC and USV did not have an LOS due to the nearby parking building. The UAV used in this measurement was the Quadcopter DJI Matrice 100. For the proof-of-concept measurement, the beam-steering configurations of the GC and UAV were precalculated and manually set into the devices. The RSS levels of the UAV directional antenna and the omnidirectional antenna on the GC were recorded to observe how the relay system works in a test environment.

While the UAV was airborne, the two antennas on the ground (USV and GC) were successfully connected and exchanged packets to each other using the relay link. The Udoo board installed on the UAV played the important role of a routing device and forwarded packets between the USB Wi-Fi dongle and Gigabit Ethernet interface connected to the MikroTik antenna.

#### 6.2.2. Measurement Results

**Figure 17:**
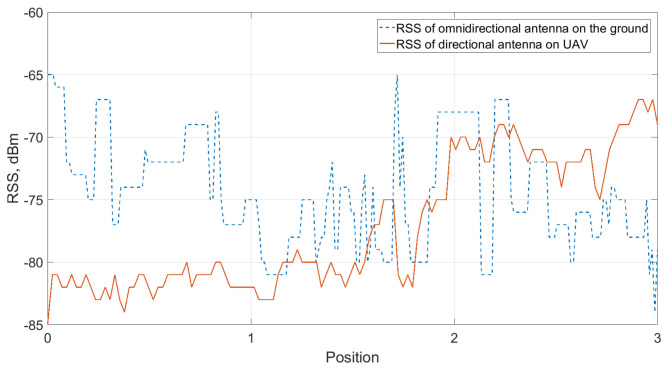
RSS level in the relay communication mode measurement.

The RSS levels at the UAV directional antenna and omnidirectional antenna on the ground are shown in Figure 17. The height of the UAV was constant at 22.5 m throughout the whole flight. There are four positions shown in Figure 17. At the beginning of the flight (position 0), the UAV was already at the height of 22.5 m above the ground. However, the directional antenna on the UAV was not aligned with the directional antenna on the GC until position 2. Before that, the links between all three parts (GC, UAV, and USV) had already been established, even though the RSS of the directional antenna on the UAV was relatively low. This was due to the fact that the distance between GC and UAV is short compared to the scenario at a lake. The RSS of the omnidirectional antenna is higher than that of the directional antenna on the UAV. As the beam started to be aligned at position 2, the signal level of the directional antenna continued increasing until position 3.

The connection between the GC and USV depends on two ground-to-air radio links—between GC and UAV, and between USV and UAV. The measurement results show that one of them (with lower RSS) will always be a bottleneck link, which limits the throughput of the overall relay system. Therefore, the position of the UAV should be well selected to optimize the bottleneck radio link.

#### 6.2.3. UAV Positioning Analysis

In commercial solutions, the UAV moves autonomously; however, the optimal relaying position still needs to be calculated. The optimal location can potentially increase the RSS levels. In this section, the simulation reflecting the RSS with varying UAV positions is discussed and evaluated.

**Figure 18:**
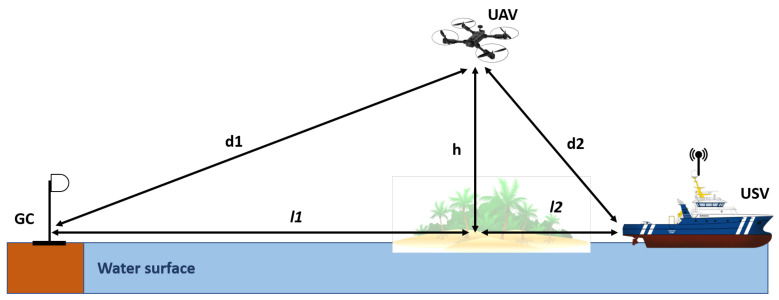
Simulation scenario for obtaining the optimized UAV position between GC and USV.

The relay scenario with the position of the UAV are illustrated in Figure 18. As discussed in Section 3.2, the channel model used to simulate the GC–UAV and UAV–USV connections is the FSPL model, since the ground-to-air radio links in this situation are in an LOS and open-space environment. In the simulations, the scenario from Section 6.1 is used as well as the parameters. The $l_{1}$ and $l_{2}$ are set to 3 km and 1 km, respectively. The UAV is assumed to fly at an altitude *h* = 22.5 m above the ground, which is higher than the average level of trees and other infrastructures on the island. The values $d_{1}$ and $d_{2}$ are calculated based on the parameters above. In the simulation, the two values $l_{1}$ and $l_{2}$ vary from 0 to 4 km, which indicates the installed location of the GC and movement of the USV. The RSS levels of the GC–UAV and UAV–USV links are calculated based on the FSPL model and then compared to each other. The one with a lower signal level is the bottleneck, which will be plotted against $d_{1}$ and $d_{2}$.

**Figure 19:**
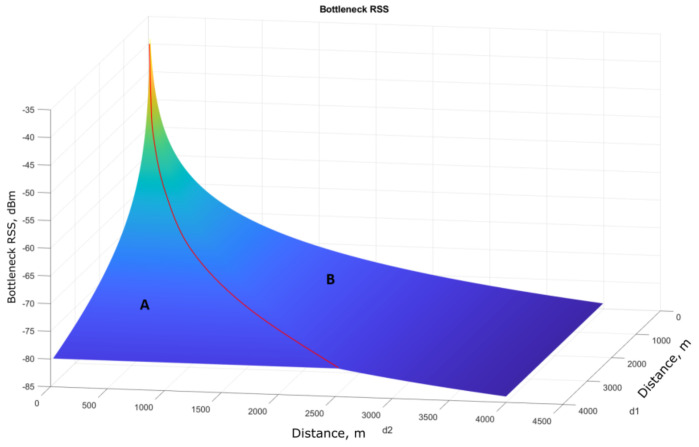
Bottleneck RSS of the overall radio link.

The bottleneck RSS plot of the communication system is shown in Figure 19. The latter shows that the RSS increases as the $d_{1}$ and $d_{2}$ distance decreases. Area A in the figure implies the cases when the bottleneck RSS only depends on $d_{1}$, regardless of the $d_{2}$ value. In area B, the RSS values are subject to the UAV–USV distance. For each constant $d_{1}$, as the distance $d_{2}$ between the UAV and the USV increases (crosses the red line), the UAV–USV link becomes the bottleneck for the overall system and the RSS values become lower than those in area A. Hence, it is necessary to keep the $d_{2}$ distance to be on the left of the red line. The red line represents the optimal RSS while the GC–UAV–USV distances are growing. To find the relation between $d_{1}$ and $d_{2}$ on that line, each $\left(d_{1},d_{2}\right)$ pair on the red line was collected and parsed to a curve fitting function in Matlab. The function polyfit() in Matlab is used for this purpose to calculate the coefficients of a polynomial that best fits the input data. Then, the line can be estimated as $d_{2}=0.6309d_{1}-0.1954$, with $d_{1}$ and $d_{2}$ measured in km. Thus, with the current antenna setup and transmitter power settings, the UAV should keep its distance to the USV to be equal to or shorter than $0.6309d_{1}-0.1954$ to optimize the overall RSS of the system. The relation for optimal $d_{1}$ and $d_{2}$ can be expressed as $d_{2}⩽0.6309d_{1}-0.1954$.

Using the estimated optimal position of the UAV against the USV, the simulated bottleneck RSS of the relay link was compared to the measured RSS.

**Figure 20:**
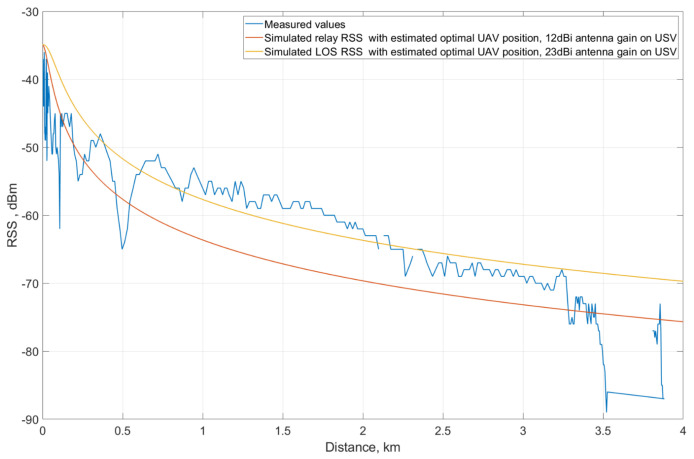
Comparison of measured RSS and simulated relay RSS.

The x-axis features the horizontal distance *l* between the GC and the USV, which means $l=l_{1}+l_{2}$. Using that, along with the height and the relation $d_{2}=0.6309d_{1}-0.1954$, a pair of $\left(l_{1},l_{2}\right)$ and $\left(d_{1},d_{2}\right)$ can be found for each distance *l*. From those values, the path loss values for GC–UAV and UAV–USV were calculated and the lower ones were collected as a basis for RSS to be plotted. Figure 20 presents the mentioned comparison. The USV loses the LOS at a distance beyond 3.5 km; the relay link (red curve) works better than the direct link (blue curve) and becomes a solution to maintain the connection between the GC and the USV. However, the direct link performs better than the relay link at distances below 3.5 km. The reason for this is the lower reception gain of the USV and UAV antennas used in the relay communication mode. In the LOS communication mode with direct GC–USV connection, the antenna on USV is directional and the gain of the main lobe is 23 dBi while in relay mode, the reception gain is 16 dBi for the directional antenna on the UAV and 12 dBi for the omnidirectional antenna on USV. The use of 12 dBi omnidirectional antenna on USV also limits the possible UAV–USV distance, which creates imbalances between optimal distance values for the relay mode components. While the UAV has to keep its proximity to the autonomous vessel, the range between the GC and the UAV becomes significantly higher than the UAV–USV distance. The 16 dBi directional antenna on the UAV cannot compensate that distance to provide an adequate signal strength compared to the 23 dBi one in the LOS mode, which leads to an unfair comparison of the LOS mode and relay mode in terms of equipment settings.

Therefore, another comparison between LOS mode and relay mode was made with equal conditions of GC and USV. Assuming that the effective radiated power from the transmitter (GC) are the same for both cases, the USV will now be assumed to use the 23-dBi directional antenna on a UAV–USV link. Using the same approach mentioned previously, the simulated relay RSS is illustrated with a yellow curve. It is clear that the relay mode system with the 23-dBi directional antenna on USV performs better than the LOS mode system when the vessel is 2-km farther than the GC. In this case, with the higher received gain in USV, the range of UAV against the USV is extended and the GC–UAV and UAV–USV links in relay mode are now more balanced in terms of optimal range.

Knowing the optimal position of the UAV in reference to the vessel provides ideas on how the UAV should be used in an autonomous collaborative offshore network. To prevent the signal loss, or session interruption in the NLOS case, the vessel should predict that there will be obstacles blocking the channel based on the map calculations and then deploy the UAV in advance to maintain the connection between the GC and the USV. After being launched, the UAV follows the vessel within a range that is calculated based on the distance between the USV and the GC using the optimal position investigated in this section. Another usage of the UAV is to extend the coverage of the wireless system. With higher USV antenna gain, the relay mode system can perform better than the LOS mode link in farther distances. Therefore, the vessel can deploy the UAV to improve the connection quality when the RSS drops below a defined value.

## 7. Conclusions

In this article, we presented the results of our research work, which aimed to design, implement, and test a wireless autonomous collaborative communication system for emerging maritime use cases. The core components of the proposed system are GC, USV, and UAV. To support offshore monitoring and unmanned operations, the designed system is based on high-speed, long-range communications between these components. Taking into account the LOS/NLOS scenarios between the GC and USV, two communication modes were defined: an LOS communication mode, which implies a direct LOS link between the GC and USV; and a relay communication mode, where a UAV is deployed to act as a relay in NLOS scenarios.

Each unmanned vehicle in the proposed autonomous collaborative system is equipped with communication modules that incorporate wireless interfaces, directional antennas, rotation mechanisms for mechanical beam-steering, and controllers for data collection and processing. Further details on the design and implementation of our communication system were provided in this article. We also assessed the performance of the designed and implemented links using measurement campaigns. Additionally, simulations were utilized—first, to compare the measurement data to analytical results based on the reviewed propagation models; second, to determine the optimal UAV position in the relay communication mode.

Potential enhancements of our communication system include conducting the measurements on the relay communication mode in a maritime environment, and developing a handover mechanism between the two defined communication modes to maintain continuous connections between the unmanned vehicles and the control station. A next step in the implementation of the overall automated offshore system depicted in Figure 1 can be the design and implementation of communication links with the AUV. Including the underwater component will enable a fully integrated collaborative system with more autonomous vehicles and further support of emerging maritime use cases.

## Figures and Tables

**Table 1:** Hardware specifications of the Beaglebone Green board.

Feature	Value
Processor	AM335x 1 GHz ARMR Cortex-A8
RAM	512 MB DDR3
On-board storage	4 GB eMMC
Accelerator support	NEON floating-point and 3D graphics accelerator
Micro USB	1, for Powering and data communication
USB	1, for Hosting
GPIO	2 ∗ 46 pin headers
Networking	1 Ethernet port
Operating temperature	0 to 75 °C

**Table 2:** Hardware specifications of the Udoo X86 board (Arezzo, Italy).

Feature	Value
CPU	Intel${}_{®}$ Pentium N3710 up to 2.56 GHz
GPU	Intel${}_{®}$ HD Graphics
RAM	8 GB DDR3L Dual Channe
Video interfaces	1∗ HDMI 1.4 (CEC), 2∗ Mini DisplayPort ++
On-board storage	32 GB eMMC soldered on-board
Networking	1∗ Gigabit Ethernet LAN interface
	1∗ M.2 Key E slot for optional Wireless Module
Audio interfaces	HD Audio Codec ALC283CG
	Microphone + Headphone Combo Connector (TRRS)
	Preamplified stereo speaker output, S/PDIF output
USB	3∗ USB 3.0 type-A sockets
Other interfaces	2∗ HSUART ports, 2∗ I2C interface, 1∗ SDIO interface
	1∗ LPC interface
Platform compability	Arduino™ 101-Compatible through standard Arduino™
	Pins layout, compatible with Arduino™ shields

**Table 3:** List of the components used in the system and their input voltages.

Category	Item	Model	Quantity	Supply Voltage [V]
Mechanical components	GC Servo motor	HS-805BB	1	4.6–6.0
	UAV Servo motor	TGY 306G-HV	1	4.8/6.0/7.2
	USV Servo motor	Dynamixel MX-28	2	12
Networking components	Directional antenna	MikroTik DynaDish 5	2	11–60
	Compact Directional antenna	MikroTik SXT 5ac	1	15–60
	Omnidirectional antenna	MikroTik GrooveA 52	1	9–30
	Router	MikroTik hEX PoE	1	12–57
Electrical components	USV and GC controller	Beaglebone Green	2	5
	UAV controller	Udoo X86	1	12
	Servo controller	Arbotix-M	1	11–12
	DC motor driver	POLOLU-713	1	2.7–5.5
	Motion sensor	Adafruit LSM9DS0	1	2.4–3.6

## Data Availability

Not applicable.
